# Interim Study: Comparison Of Safety And Efficacy of Levofloxacin Plus Colistin Regimen With Levofloxacin Plus High Dose Ampicillin/Sulbactam Infusion In Treatment of Ventilator-Associated Pneumonia Due To Multi Drug Resistant Acinetobacter

**Published:** 2018

**Authors:** Reza Mosaed, Mehrdad Haghighi, Mehran Kouchak, Mir Mohammad Miri, Sara Salarian, Seyedpouzhia Shojaei, Abdolreza Javadi, Saeed Taheri, Pardis Nazirzadeh, Masoumeh Foroumand, Mohammad Sistanizad

**Affiliations:** a *Department of Clinical Pharmacy, school of pharmacy, Shahid Beheshti University of Medical Sciences, Tehran, Iran. *; b *Department of Infectious Diseases, Imam Hossein Teaching and Educational Center, Shahid Beheshti University of Medical Sciences, Tehran, IR Iran. *; c *Department of Critical Care Medicine, Imam Hossein Medical and Educational Center, Shahid Beheshti University of Medical Sciences, Tehran, Iran. *; d *Imam Hossein Teaching and Educational Center, Shahid Beheshti University of Medical Sciences, Tehran, Iran.*; e *Department of pharmacoeconomics and pharma management, school of pharmacy, Shahid Beheshti University of Medical Sciences, Tehran, Iran.*

**Keywords:** Pneumonia, Ventilator-Associated, Acinetobacter, Ampicillin-Sulbactam, colistin, Levofloxacin

## Abstract

Due to the emerging antibiotic resistance of Acinetobacter, which is the leading cause of ventilator-associated pneumonia (VAP) in critically ill patients, there is an urgent need for studies comparing various antibiotic regimens for its treatment. In this single blinded randomized clinical trial, adult patients with VAP due to multi drug resistant Acinetobacter (MDRA), were randomly assigned to receive 9×10^9 ^unit loading dose of colistin followed by 4.5×10^9^ unit intravenously twice daily plus 750 mg intravenous levofloxacin daily or continuous infusion of ampicillin/sulbactam, 24g daily plus 750mg IV levofloxacin daily. Dose and dosing interval were adjusted according to serum creatinine levels during the study. Clinical and microbiological cure, inflammatory biomarkers, and possible adverse effects were recorded in participants. Twenty-nine patients were recruited (14 in colistin and 15 in ampicillin/sulbactam groups). Three patients were excluded in each group. Clinical response occurred in 3 (27%) and 10 (83%) in colistin and ampicillin-sulbactam arms, respectively (P = 0.007). Nephrotoxicity happened in 6 (54%) and 1 (8%) of cases in colistin and ampicillin-sulbactam groups, (P = 0.016). 14-day and 28-day survival rate were significantly higher in ampicillin-sulbactam group compared to colistin arm with P values of 0.002 and 0.049, respectively. This study revealed that levofloxacin plus high dose ampicillin/sulbactam as continuous infusion is more effective than levofloxacin plus colistin in patients with MDR Acinetobacter VAP with significantly lower risk of nephrotoxicity.

## Introduction

Approximately 10 percent of patients who are mechanically ventilated in intensive care unit (ICU) are prone to ventilator-associated pneumonia (VAP), which is associated with increased length of ICU stay and mortality rate (1). Several microorganisms could cause VAP. In Asian countries, main organisms that isolated in VAP cases are Acinetobacter spp., P. aeruginosa, S. aureus, and K. pneumonia (2).

Acinetobacter, which is the leading cause of VAP in critically ill patients (3), is a nonfermenting, gram-negative, aerobic coccobacillus belonging to the Moraxellaceae family (4-6). Due to the emerging antibiotic resistance of Acinetobacters (7), high profile antimicrobials and combination regimens can be used as a treatment approach in MDR infections (8). 

The treatment recommendation according to the Infectious Diseases Society of America/American Thoracic Society (IDSA/ATS) guidelines (2016), are carbapenem or ampicillin/sulbactam or intravenous polymyxin (colistin or polymyxin B) if gram-negative bacilli are susceptible to only polymyxins (9).

Among studies in Iran, Alavi-Moghadam *et al*, had shown that All of A. baumannii isolates from VAP patients in Imam Hussein hospital in Iran were resistant to imipenem (10), therefor the last choice of treatment would be colistin that has several limitations. According to one study, rates of AKI from colistin use at 48 h and 7 days of its administration were 12% and 29% respectively (11). Beside its high rate of nephro- and neurotoxicity which limits its clinical use (reference) another limitation is colistin shortage in several countries (12). Therefore, finding an alternative regimen to colistin could be detrimental in some conditions. Furthermore, the IDSA/ATS guidelines (2016) declare an urgent need for studies comparing various antibiotic regimens in the treatment of pneumonia due to Acinetobacter species (9).

Primary objective of the current study is to compare efficacy and safety of continuous infusion of high dose ampicillin/sulbactam with colistin for treatment of VAP due to MDR Acinetobacter.

*Materials and Methods*

This single blind randomized clinical trial (RCT) was conducted at a 30-bed medical-surgical intensive care unit of Imam Hussein medical center, a 600-bed hospital affiliated to Shahid Beheshti University of Medical Science (SBMU) in Tehran, Iran. This study has been approved by institutional review boards of ethics committee of SBMU (IR. SBMU.PHNM.1396.889), and has been registered in Iranian registry of clinical trials, too. (IRCT20120703010178N15)

Pneumonia was defined as radiographic appearance of a new and persistent pulmonary infiltrate and two of the following criteria: temperature of >38 ºC or <35.5 ºC, leukocytosis (leukocyte count, > 12,000 cells/mm3) or leukopenia (leukocyte count, <4000 cells/mm3), decline in oxygenation (O2 saturation < 90 %), the presence of purulent bronchial secretions, and increased amount of purulent sputum (9). 

If sputum samples gram’s stains showed at least 25 neutrophils and less than 10 epithelial cells per low-power field, they would be sent for bacterial culture. If the sputum culture had an at least moderate growth, etiologic pathogen of pneumonia would be determined.

The hospital’s microbiology laboratory determined the antimicrobial susceptibilities for isolates by the E-test method (High media®) based on CLSI guideline (REF). The severity of illness was evaluated by the APACHE II score on the basis of the worst data point of the 24 h before entering to study (13).

Inclusion criteria were defined as mechanical ventilation for > 48 h, not documented infection before the initiation of mechanical ventilation, Acute Physiology and Chronic Health Evaluation (APACHE) II score of more than 8, Clinical Pulmonary Infection Score (CPIS) ≥ 6 and positive endotracheal tube (ETT) culture of MDR Acinetobacter isolate resistant to at least three classes of antimicrobial agents as below: all penicillin’s and cephalosporin’s (including inhibitor combinations), fluoroquinolones, and aminoglycosides.

The patients were excluded if they had history of moderate or severe hypersensitivity reactions to beta-lactam antibiotics or colistin, kidney injury defined as GFR<60mL/min (Before entering the study) and GFR <30mL/min (day 1 to 3 of the study), receiving antibiotics for this episode of VAP for more than 96 h before study medication administration, co-infection in another organ, acute respiratory distress syndrome, chest trauma with fracture of the sternum, ribs, or both, lung cancer within the last 2 years, chronic bronchitis with an increase in severity within the last 30 days, tuberculosis on treatment, suspected atypical pneumonia, cystic fibrosis, and severe burns to greater than 15% of the body surface area.

Written consent was obtained from patients or their family. The patients were allocated in colistin or ampicillin sulbactam arm using block randomization. The patients in colistin group received 9×10^9 ^unit loading dose of colistin followed by 4.5×10^9^ unit intravenously twice daily plus 750mg intravenous levofloxacin daily. Subjects in ampicillin-sulbactam group received continuous infusion of ampicillin/sulbactam 24g daily (6g IV ampicillin/sulbactam (at a ratio 2:1) four times a day, each dose infused over 6 h) plus 750mg IV levofloxacin daily. Dose and dosing interval was adjusted according to the serum creatinine levels. The following variables were recorded for every patient enrolled in this protocol: age, sex, APACHE II score of the day entering patient to study, dates of admission, and discharge from the ICU and the hospital, comorbidities (i.e., COPD, trauma, diabetes mellitus and cardiovascular disease), and duration of mechanical ventilation. ICU physician considered clinical response, laboratory and imaging of the patient determined during the treatment.

The primary outcome measure was the clinical cure of VAP. Infection was considered to have been cured if there was remission of pneumonia-related symptoms. As secondary end points, we evaluated microbiological cure time and mortality in 14 and 28 days. Microbiological eradication was considered to have been occurred if the aspirate culture was negative for Acinetobacter. If the culture result was positive but the patient was clinically cured, the treatment would stop. Renal function was monitored by daily measurement of the serum creatinine level. Renal failure was defined as a 30% increase in serum creatinine.

*Statistical Analysis*

All statistical analyses were performed using the SPSS for Windows (Version 21.0; SPSS Inc., Chicago, IL, USA). Categorical variables were compared using χ2 test or Fisher exact test, as appropriate. Continuous variables were tested for normality of distributions by Kolmogorov–Smirnov test, and then compared by Student’s t-test or the Mann-Whitney U test, as appropriate. All the tests were two-tailed, and a P value of < 0.05 was considered significant.

## Results

**Figure 1:**
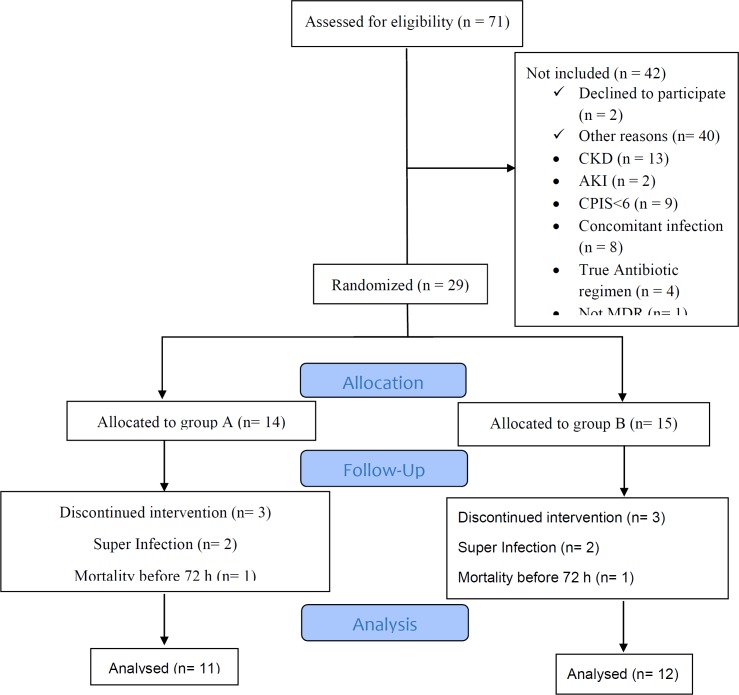
Disposition of MDRA VAP patients included in the analysis of the impact of levofloxacin/Colistin and levofloxacin/high dose ampicillin-sulbactam infusion

MDRA, multidrug-resistant Acinetobacter; VAP, ventilator associated pneumonia; CKD, chronic kidney disease; AKI, acute kidney injury; CPIS,clinical pulmonary infection score; ARDS, acute respiratory distress syndrome;

From September 2017 through May 2018, 1091 patients were admitted to ICU. Seventy-one patients with sputum culture of Acinetobacter were assessed, which 42 of them did not meet inclusion criteria. Details are showed in Figure 1. From the remaining 29 patients, 14 were allocated in colistin and 15 in ampicillin-sulbactam arms. For all participants, the demographic and other related data including age, sex, comorbidity, APACHE II score, and CPIS were recorded. As demonstrated in table-1, baseline characteristics were similar in two arms of the study. 

**Table 1:** Baseline characteristics

**Characteristic**	**Colistin group (11)**	**Ampicillin-Sulbactam group (12)**	***P*** **-value**
**Age**	66.9 ± 9.91	64 ± 23.46	0.70
**Male gender**	7 (63%)	6 (50%)	0.51
**Comorbidity (at least one)**	8 (72.72%)	9 (75%)	0.75
**Truma**	1 (9.09%)	3 (25%)	
**Diabetes Mellitus**	1 (9.09%)	0	
**Cardiovascular**	1 (9.09%)	2 (16.66%)	
**Neurological diseases**	2 (18.18%)	1 (8.33%)	
**Poly comorbidities**	3 (27.27%)	3 (25%)	
**No comorbidity**	3 (27.27%)	3 (25%)	
**APACHE II (0)**	19.9 ± 3.38	18.41 ± 4.12	0.37
**CPIS (0)**	9 ± 1.24	9.41 ± 1.31	0.45

APACHE II, acute physiologic and chronic health evaluation; CPIS, clinical pulmonary infection score.

**Table 2:** Outcome

**Outcome**	**Colistin group (11)**	**Ampicillin-Sulbactam group (12)**	***P*** **-value**
**Length of stay in**			
**Hospital**	41.9 ± 34.02	35.41 ± 10.49	0.53
**ICU**	22.09 ± 7.5	25.16 ± 10.83	0.44
**MVT**	25 ± 16.99	22 ± 11.69	0.65
**All-cause Mortality**			
**28 days**	9 (81.81%)	5 (41.66%)	0.04
**14 days**	8 (72.72%)	1 (8.33%)	0.002
**Clinical response**	3 (27.27%)	10 (83.33%)	0.007
**AKI**	6 (54.54%)	1 (8.33%)	0.016

ICU, Intensive Care Unit; MVT, mechanical ventilation time period; AKI, acute kidney injury

**Figure 2:**
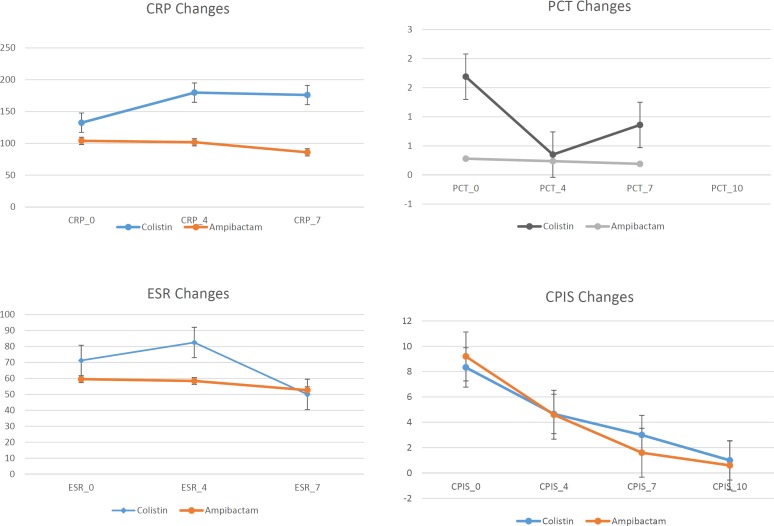
Acute phase reactants (APR) and CPIS changes in patients who have cured

CRP: C-reactive protein; PCT: procalcitonin; ESR: erythrocyte sedimentation rate; CPIS: clinical pulmonary infection score.

Three patients were excluded in each group because the second sputum culture revealed microorganism other than Acinetobacter in follow up culture at 4^th^ day in two cases in each group (both in colistin group were providencia and in ampicillin-sulbactam group, one of them was providencia and another was P. aeruginosa) and one patient in each group was expired in less than 72 h after inclusion in the study. Finally, 11 patients in the colistin group and 12 in the ampicillin-sulbactam group entered to the analysis. 

Clinical cure with successful discontinuation of antibiotics happened in 3 (27%) and 10 (83%) in colistin and ampicillin-sulbactam arms, respectively (P = 0.007). In colistin and ampicillin-sulbactam groups, mean treatment duration was 8 (range, 7 to 9 days) and 9.1 days (range, 6.9 to 11.3 days), respectively. Surveillance culture on 4^th^ day of the study showed microbiological eradication in all subjects in colistin arm and in 8 cases in ampicillin-sulbactam group. 

Nephrotoxicity happened in 6 (54%) and 1 (8%) of cases in colistin and ampicillin-sulbactam group, respectively (P = 0.016). In colistin group mean time for occurrence of nephrotoxicity was 3.43 ± 1.4 days. Nephrotoxicity in one patient in ampicillin-sulbactam group happened at seventh day of the study. 

As presented in table-2 the 14-day and 28-day survival rates were significantly higher in ampicillin-sulbactam group compared to colistin arm with P values of 0.002, and 0.049, respectively. Length of hospital (P = 0.53) and ICU (P = 0.44) stay was similar between two groups. In addition, the differences in duration of mechanical ventilation did not reach statistical significance (P = 0.65).

Figure 2 reveals data related to inflammatory biomarkers including PCT, CRP, ESR, and CPIS as scoring system for evaluating pneumonia in the patients with successful clinical response to treatment including 3 patients in colistin and 10 in ampicillin/sulbactam arms of the study. According to Figure 2, the trend of CPIS and ESR was decreasing but CRP and PCT changes didn’t show a reasonable trend.

## Discussion

The most important outcome of this research is intravenous ampicillin/sulbactam plus levofloxacin regimen that is more effective and safer than intravenous colistin plus levofloxacin in MDR VAP patients due to Acinetobacter. Several studies have evaluated the efficacy of high dose ampicillin/sulbactam in MDRA infection treatment. Betrosian *et al* had run a prospective cohort study in adult critically ill patients with VAP, and mentioned that colistin and high-dose ampicillin/sulbactam were comparably safe and effective treatments for critically ill patients with MDR A. baumannii VAP. The clinical success rate was 76.9 % for ampicillin/sulbactam group and 73.3% for colistin group (14). However, this study represents more superiority of ampcillin/sulbactam (83.33%) vs colistin (27.27%) in combination with levofloxacin. This variance could be due to three main reasons, first using high dose ampcillin/sulbactam, second administration of combination with levofloxacin, and third administration of ampcillin/sulbactam as continues infusion. Beom jeong *et al*, reported high dose sulbactam treatment for VAP caused by carbapenem-resistant A. baumannii (CRAB). In the study, 32 vials of ampicillin-sulbactam (16 g ampicillin, 8 g sulbactam) were administered per day. In 65.5% of patients they observed clinical improvement so they conclude that High-dose sulbactam therapy may be more effective for the treatment of CRAB-VAP (15). An *in-vitro* study conducted in Kashan, has shown the combination of levoﬂoxacin plus a bactericidal antibiotic such as ampicillin/sulbactam have synergistic effects on MDRA (FIC index:≤ 0.5). This combination was effective in 90% of the MDRA isolates (16). Joel M. Dulhunty *et al* in a multicenter double-blind, randomized controlled trial published that continuous administration of beta-lactam antibiotics achieved higher plasma antibiotic concentrations than intermittent administration with improvement in clinical cure (17). A Systematic Review with E. Falagas and colleagues showed that mortality was lower among the patients receiving extended or continuous infusion of carbapenems or piperacillin/tazobactam compared to those receiving short-term (18). However, no published study was found for evaluating ampicillin/sulbactam continues infusion because its IV infusion solutions just will be stable in NS for 8 h and it could not be used for 24 h infusion period (19). To solve this problem, we administered ampicillin/sulbactam as 6g (4:2) every six hours and each dose was infused over six hours.

In this study the higher rate of nephrotoxicity was observed in the colistin group (54.54%). In a Prospective Cohort Study that evaluated colistin-associated Acute Kidney Injury in Severely Ill Patients, from 70 patients who received colistin at a median daily dose of 9 million IU, (44%) developed AKI (12). This difference with our study data is might be due to our smaller population or to using the regimen concomitant with levofloxacin.

colistin nephrotoxicity usually occurs within the first week of use. Age, severity of the underlying disease, co-existence of septic shock, use of other nephrotoxic agents, time of exposure to colistin and cumulative dose are reported to be associated with colistin nephrotoxicity (11, 12, 20).

Based on our knowledge, there is no study that compares the efficacy of new regimen with colistin for treatment of MDR Acinetobacter VAP with culture just sensitive to colistin. Ventilator associated pneumonia caused by extensive-drug resistant Acinetobacter species, that colistin is the remained choice, have been reported in some studies (21, 22). Because of colistin shortage and its high probability of AKI, we decided to evaluate new regimen for substituting with colistin. 

Our results demonstrated that CPIS decreased in the patients who had clinical response, in accordance with IDSA/ATS guidelines (2016) recommendation on using this score for discontinuation of antibiotic therapy (9). Parallel to CPIS, ESR decreased in these patients, too. Markanday *et al*, in a review about role of acute phase reactants (APR) in 2015 concluded that the ESR rises within 24–48 of the onset of inflammation and drops gradually with resolution of inflammation. Also it is not specific for infection, its decrease reveals resolution of any type of inflammation including infection and could guide clinicians for discontinuing antibiotics (23).

This study has encountered with some limitations. First, the study was single-centered, it was an interim study and the patients’ population was small but because of high mortality rate in colistin group we decided to stop the study. Preparing infusion pump for all the patients in this economic situation was another problem. After few months empiric, the use of ampicillin/sulbactam by physicians was increased because the colistin had shortage and the clinical success of ampicillin/sulbactam plus levofloxacin regimen was wonderful.

## Conclusion

In conclusion, the result demonstrated that the treatment with levofloxacin plus high dose ampicillin/sulbactam is superior to one with levofloxacin plus colistin in the patients with MDR Acinetobacter ventilator associated pneumonia. In addition, the rate of acute kidney injury was significantly lower in ampicillin/sulbactam group compared to colistin arm of the study.
